# Patterns of fatty acid usage in two nocturnal insectivores: the Mediterranean house gecko (*Hemidactylus turcicus*) and the Etruscan pygmy shrew (*Suncus etruscus*)

**DOI:** 10.1242/jeb.245963

**Published:** 2023-10-11

**Authors:** Shahar Dubiner, Amit Kashi, Ariel Drabkin, Pablo Blinder, Eran Levin

**Affiliations:** ^1^ School of Zoology, Faculty of Life Sciences, Tel Aviv University, Tel Aviv 6997801, Israel; ^2^ School of Neurobiology, Biochemistry and Biophysics Faculty of Life Sciences, Tel Aviv University, Tel Aviv 6997801, Israel; ^3^ Sagol School for Neuroscience, Tel Aviv University, 6997801, Israel

**Keywords:** Circadian rhythm, Diet, Fatty acid, Nutrient oxidation, Stable isotope, Unsaturation, *Suncus etruscus*, *Hemidactylus turcicus*

## Abstract

Dietary fatty acids (FAs) have been demonstrated to be differentially stored or used as a metabolic fuel, depending on carbon chain length or saturation level. However, intestinal absorption also differs among FAs, potentially biasing conclusions on functional differences and their subsequent implications. We tested dietary FA usage in a nocturnal insectivorous reptile and a nocturnal insectivorous mammal of similar size: the gecko *Hemidactylus turcicus* and the shrew *Suncus etruscus*. We compared the relative presence of ^13^C isotopes in breath and feces following ingestion of three isotopically enriched fatty acids: linoleic acid (a polyunsaturated FA), oleic acid (monounsaturated) and palmitic acid (saturated). Both species oxidized linoleic and oleic acids at much higher levels than palmitic acid. Egestion of palmitic acid in feces was much higher than that of linoleic and oleic acids. The major difference between geckos and shrews was that the latter digested fatty acids much faster, which was best explained by the difference in the metabolic rates of the species. Circadian differences were evident for gecko metabolic and FA oxidation rates, peaking at night; for shrews, peak oxidation was achieved faster at night but rates did not differ. Our study is among the first to integrate oxidation and absorption patterns, as well as metabolic rates and their rhythms, providing important insights into the utilization of different dietary FAs in different species.

## Teaser

**Summary:** Oxidation and egestion, metabolic rates and their rhythms in house geckos and pygmy shrews fed with three isotopically enriched fatty acids.

## INTRODUCTION

Fatty acids (FAs) constitute part of the major macromolecules essential to all life. FAs serve many functions, from building the cell membrane structure and signaling, to energy reserves and metabolic fuel. FAs can be obtained from the food or synthesized *de novo* (De Carvalho and Caramujo, 2018). They are divided into three major categories according to the number of double bonds they have: saturated, monounsaturated and polyunsaturated fatty acids (SFAs, MUFAs and PUFAs, respectively). Of these, some PUFAs are considered essential in vertebrates, including reptiles and mammals, meaning that they must be acquired from the diet (Hulbert, 2003). FAs used for metabolism are converted to free fatty acids, whereupon they undergo β-oxidation and enter the Krebs cycle as acetyl-CoA. Downstream, ATP is produced and the first carbon atom of the FA chain (C_1_) is released as CO_2_ (Price, 2017). Isotopic labeling of that carbon has been used in various animal and human studies to show differences in oxidation between different FAs (Krishnan and Cooper, 2014; Leyton et al., 1987; McCue et al., 2010; Rosner and Voigt, 2018; Seltzer et al., 2023). MUFAs and PUFAs are oxidized at higher rates than long-chain SFAs such as palmitic acid. Some studies infer that lower oxidation of SFAs indicates higher allocation to tissues relative to other FAs (e.g. DeLany et al., 2000). Other studies report an influence of dietary FA profiles on body composition or physiology in both reptiles (Simandle et al., 2001) and mammals (Ayre and Hulbert, 1996; Benítez et al., 2015; Ruf et al., 2006; Thomson et al., 1989).

Differential FA oxidation or allocation in tissues, however, and any inference regarding their downstream effects on physiology or health, cannot be taken in isolation from the differential absorption of macromolecules in the digestive tract. The abovementioned studies, and others taking similar approaches, assume explicitly (e.g. DeLany et al., 2000; Plasman et al., 2019) or implicitly, that different FAs are energetically equivalent. Uptake of free fatty acids, phospholipids and triglycerides by the intestine follows different pathways (Price, 2017) and they may therefore be absorbed at different rates when ingested in different forms. Moreover, FA characteristics, such as length and saturation level, may determine uptake efficiency. Shorter FAs are more soluble in the intestinal lumen and therefore reach the mucosa layer more easily (Decker, 1996; Ramírez et al., 2001). More highly saturated FAs were less efficiently absorbed when fed to mammals (Tomarelli et al., 1968) owing to their linear structure forming insoluble solids in the basic environment of the duodenum (Ramírez et al., 2001). Even when rat intestine tissue *in vitro* showed similar mucosal uptake between an SFA and a PUFA, FA esterification (and therefore, the possibility for their transport to other tissues as a triglyceride) was lower for the SFA (Ockner et al., 1972). In contrast, studies of avian models, such as chickens (Noyan et al., 1964; Tancharoenrat et al., 2014) and sparrows (McCue et al., 2011) did find relatively high levels of SFA absorption and allocation.

Some studies explain differential usage of FAs, asserting that some are better as metabolic fuel (DeLany et al., 2000) or are more efficiently utilized during fasting (Rosner and Voigt, 2018). In postabsorptive animals, stored FAs are oxidized for energy – part of which can be used to digest the next meal (McCue et al., 2015). Reptiles, in particular, exhibit a prolonged peak in metabolic rate after they eat [termed specific dynamic action (SDA): McCue, 2006; Secor, 2009] since digestion of prey requires an energetic investment. The partitioning of this investment between the dietary and stored energy is uncertain (McCue et al., 2005, 2015). Despite high SDA in reptiles, their overall resting metabolic rates are much lower than in similar-sized endotherms (Hulbert, 2003; Nagy, 2005). This enables reptiles to feed less frequently and have slower digestion than similar-sized endotherms (Price, 2017) with equivalent diets. Slower digestion improves the efficiency of nutrient assimilation (McConnachie and Alexander, 2004; Karasov and Martínez del Rio, 2007), potentially resulting in different patterns of fatty acid usage.

Here, we aimed to compare for the first time the use of different FAs from the diet, integrating oxidation and absorption between an insectivorous reptile and an insectivorous mammal of similar body mass. The Mediterranean house gecko (*Hemidactylus turcicus*) is nocturnal and insectivorous (Bar et al., 2021) with a mean mass of 2.1 g (data from the Steinhardt Museum of Natural History). Its natural distribution extends from around the Mediterranean and Arabia to India and can be found across Israel (Bar et al., 2021). The Etruscan pygmy shrew (*Suncus etruscus*) is a nocturnal and insectivorous mammal (Arbel, 2008) with a mean mass of 1.8 g (data from the Steinhardt Museum of Natural History), whose distribution extends from around the Mediterranean and Arabia to Southern Asia (Alekseev and Sheftel, 2017) and can be found across Israel (Arbel, 2008). Its mass-specific basal metabolic rate is the highest of any mammal (Jürgens et al., 1996). We tracked different ingested fatty acids through absorption and oxidation in order to identify the differences and similarities in their metabolism in two different vertebrate classes.

## MATERIALS AND METHODS

### Animal collection and husbandry

We collected Mediterranean house geckos [Squamata: Gekkonidae: *Hemidactylus turcicus* (Linnaeus 1758)] by hand, in and around Tel Aviv University's Botanical Garden (32.11°N, 34.81°E) and at a nearby natural area (32.13°N, 34.80°E). Geckos were acclimated at 27–28°C for 2–4 weeks in separate terraria (302×196×147 mm) located in the Meier Segals Garden for Zoological Research in Tel Aviv, with shelter and water *ad libitum* and fed with crickets (*Acheta domesticus*) 2–3 times a week. Terraria were exposed to an artificial light with a 12 h photophase from 06:00 to 18:00 h (ignoring daylight saving time). To ensure the animals were postabsorptive when ingesting the ^13^C-enriched cricket, each individual was fasted for 4–6 days before the respirometry trials. The animals were fed again following the end of the experiment and released at their capture site.

We collected Etruscan pygmy shrews [Eulipotyphla: Soricidae: *Suncus etruscus* (Savi 1822)] in pitfall traps in a field at Ramat Sirin (32.65°N, 35.52°E). Shrews were moved to a breeding colony at the Meier Segals Garden, where they were kept for 7 months, in separate terraria (400×600×330 mm) at 27–28°C with shelter and water *ad libitum*, and fed daily with crickets and mealworms. Terraria were exposed to artificial light matching the natural daylight hours. The animals were fed again following the end of the experiment and returned to their colony at the zoo.

Geckos were collected under permit #2021/42875 and shrews under permit #2020/42627 from the Israel Nature and Parks Authority. Geckos were tested under permit #22101662 and shrews were tested under permit #0230421 from Tel Aviv University's Ethics Committee.

### Feeding and respirometry trials

At the start of each trial, animals were fed a cricket (*A. domesticus*) treated with one of three ^13^C_1_ isotopically enriched fatty acids (FAs): linoleic acid (LA; a PUFA), oleic acid (OA; a MUFA) or palmitic acid (PA; an SFA), purchased from Cambridge Isotope Laboratories, USA. OA and LA were injected into crickets (1 μl in live crickets for geckos, 5 μl in dead crickets for shrews), whereas an equivalent amount (in mol) of PA (roughly 0.8 mg for geckos and 4.1 mg for shrews) was melted at 65°C and applied to the cricket's thorax externally. To assess the β-oxidation patterns of each FA, we placed the animals in a sealed and darkened metabolic chamber (50 ml for geckos and 850 ml for shrews) kept at 25–26°C in a pull-mode respirometry setup (Urca et al., 2021). Room air was pulled at a constant rate (30 ml min^−1^) through an ascarite column (CO_2_ absorbent) into the chamber and then directly into a G2121-i cavity ring-down spectroscopy (CRDS) stable carbon isotope analyzer (Picarro, Santa Clara, CA). The CRDS was calibrated routinely with validated standards. The ^13^CO_2_ and ^12^CO_2_ contents in the exhaled breath were measured continuously for at least 72 h for geckos and 12 h for shrews from which δ^13^C (expressed in δ^13^C VPDB; Werner and Brand, 2001) was calculated. Geckos were fed at different times (ranging from 08:00 to 21:00 h, see Table S1), to enable us to disentangle the effects of circadian rhythms from those of digestion timing; gecko sample sizes were 5 for LA, 7 for OA (three of which shed their skin during the measurement) and 6 for PA (one of which had little to no oxidation); a different individual was used for each trial. Shrews were each measured once during the day (beginning at 06:00 h) and again on a separate occasion during the night (beginning at 18:00 h) for each FA. That is, the treatment was repeated so that each individual shrew was treated with each FA at both day and night (at least a week apart between treatments). Sample size was 7 for all treatments except the night PA treatment (*n*=6).

### Egestion of fatty acids in feces

Individuals of both species were fed crickets treated with one of the three ^13^C_1_ isotopically enriched FAs, as detailed above (*n*=5 for all treatments, except geckos fed LA, for which *n*=4). We gathered, dried and homogenized all feces from the metabolic chamber (and as control, from the animal's terraria), after which 1 mg of each animal's feces (weighed with analytic scales with a precision of 0.01 mg) was loaded into individual tin capsules. The δ^13^C for each sample was measured using a Picarro G2121-i cavity ring-down spectroscopy (CRDS) ^13^C stable isotope analyzer with an A0201 combustion module (Santa Clara, CA; as described in Levin et al., 2016). A validated standard of sugar was used every 10 samples to ensure analyzer accuracy.

### Statistical analyses

To determine the effect of FA feeding treatment on gecko metabolic rate, we used a linear model with FA as a fixed factor and *V̇*_CO_2__ as the dependent variable. Because the same individual shrews were measured in all the treatments, we used a linear mixed model with FA as a fixed factor and *V̇*_CO_2__ as the dependent variable, and shrew identity as a random factor. To compare the two species, we used a linear model with species as an additional fixed factor. The period of *V̇*_CO_2__ oscillation (τ) with the time of day (between 20:00 h on the first day to 20:00 h on the third day, a timeframe when all geckos were measured) was determined for geckos using the ‘lsp’ function (Lomb–Scargle periodogram) implemented in the R package ‘lomb’ (https://CRAN.R-project.org/package=lomb). This was tested for log *V̇*_CO_2__ of all individuals together except the three geckos that shed their skin during the measurement, because shedding influences their metabolism (Vardi et al., 2023). The amplitude and acrophase (cycle peak point, θ) of *V̇*_CO_2__ oscillations were calculated given the above τ value using the ‘cosinor’ function (cosinor-based rhythmometry; Cornelissen, 2014) implemented in the R package ‘card’ (https://CRAN.R-project.org/package=card).

To analyze the results of the FA feeding treatments, we used generalized linear models (GLMs, Gamma family with a log link) for geckos and generalized linear mixed models (GLMMs, with shrew identity as a random factor, gamma family with a log link) for shrews. Since the δ^13^C data contained non-positive values, the relevant GLMs and GLMMs were fitted after subtracting baseline δ^13^C from all values. To determine the differences in FA usage as metabolic fuel, we designated FA as a fixed factor and the maximum δ^13^C values in the breath as the response variable. In shrews, day/night treatment was used as an additional fixed factor (since they were fed and measured once at night and once in the morning, whereas gecko measurements spanned several days). To determine the differences in FA egestion between FAs, we modelled FA as a fixed factor and the feces δ^13^C values as the response variable. To determine the effect of FAs on the length of time until free fatty acids were depleted, we modeled FA (and in shrews, day/night) as the predictors and peak duration as the response (i.e. the time peak δ^13^C values until δ^13^C values decreased to 25% of their maxima). In an additional model, we and modelled FA (LA and OA only, since PA has very low oxidation rates) and log mean *V̇*_CO_2__ as fixed factors, with peak duration as the response. To determine the effect of treatment and metabolic rate on the time required to reach the peak of FA oxidation, we modelled FAs and log mean *V̇*_CO_2__ as fixed predictor factors (and in shrews, day/night) and the time until maximum δ^13^C as the response. Comparison of the two species was done using GLMs, with a similar structure as the GLMs for geckos but adding species as a fixed factor. Estimated *R*^2^ values (*R*^2^_est_) for the mixed models were estimated by calculating *R*^2^ for the same model without the random factor. Statistical analyses were performed in R 4.2.2 (https://www.r-project.org/).

## RESULTS

**Fig. 1.**
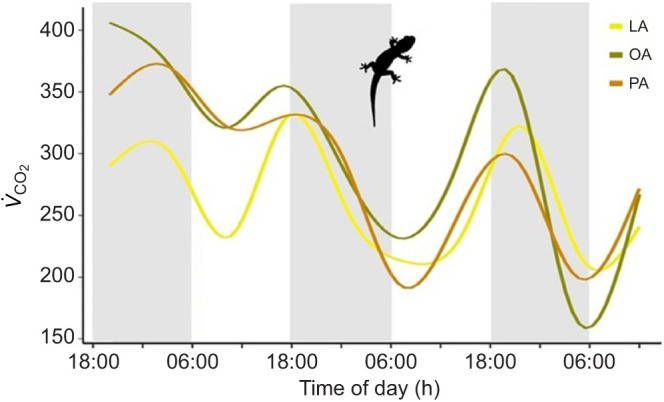
**Circadian oscillations of** ***V**˙***_CO_2__ (µl min^−1^) in geckos for all three fatty acid groups (generalized additive model).** Period is 23.96 h (*P*<0.001), amplitude is 52 µl h^−1^ and acrophase is at 19.3 h. The animals were measured in full dark, but their acclimation photophase was at 06:00–18:00 h (light background). Three days of measurement are presented, restricted to the hours during which all individuals (*n*=14, three shedding geckos not shown) were measured. LA, linoleic acid; OA, oleic acid; PA, palmitic acid.

Mean body mass was 2.5±0.2 g (mean±s.e.m. for the *n*=8 individuals weighed) for geckos and 2.8±0.2 g for shrews (*n*=7). *V̇*_CO_2__, indicative of whole body metabolic rates, did not differ between the FA groups and was 3.7 times higher in shrews (1111±47 µl h^−1^) than in geckos (300±26 µl h^−1^; *P*<0.001, model *R*^2^=0.59). Gecko *V̇*_CO_2__ exhibited a clear circadian rhythm despite all animals being kept in total darkness, with a period (τ) of 23.96 h (Lomb–Scargle: *P<*0.001; Fig. 1), an amplitude of 52 µl h^−1^ and an acrophase (θ) at 19.3 h (early in the subjective scotophase, beginning at 18:00 h; Fig. 1). In the three geckos that shed their skins during measurement, circadian rhythms were not detected. Shrew *V̇*_CO_2_ _did not differ between the night and day measurements (*P*=0.192).

**Fig. 2.**
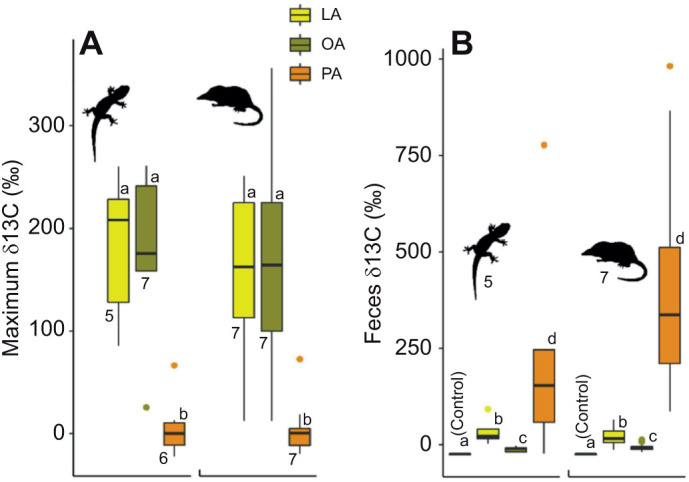
**Patterns of oxidation and egestion of FAs in geckos and shrews.** (A) For both species, OA and LA were equally oxidized as evidenced by maximum δ^13^C levels, whereas PA was oxidized at very low levels. (B) δ^13^C in the feces was higher for all three FA groups compared with the control but highest for PA. Letters indicate statistically significant differences.

In geckos, the average maximum level of ^13^C isotopically enriched CO_2_ in the breath from oxidation of PA (δ^13^C=7‰, 95% CI: [–1,17]) was lower than that of OA (δ^13^C=180‰ [120,265]; *P*<0.001) and LA (δ^13^C=181‰ [117,277]; *P*=0.002; R^2^=0.54; Fig. 2A). In shrews, the maximum δ^13^C of PA (δ^13^C=0‰ [–4,6]) was also lower than that of OA (δ^13^C=153‰ [118,196]; *P*<0.001) and LA (δ^13^C=154‰ [118,197]; *P*<0.001; *R*^2^_est_=0.36; Fig. 2A). Maximum δ^13^C and was 6.8% higher in the day trials than in the night trials (*P*<0.001). The two species did not differ in maximum δ^13^C for any FA (*P*=0.47). The δ^13^C of gecko feces was higher for PA (δ^13^C=242‰ [198,369]) than for LA (δ^13^C=35‰ [11,100]; *P*=0.007), which was, respectively, higher than for OA (δ^13^C=−11‰ [–18,–2]; *P*=0.012) and all were higher than the control group (δ^13^C=−24‰ [–26,–21]; *P*=0.008; *R*^2^=0.18; Fig. 2B). The δ^13^C of shrew feces was higher for PA (δ^13^C=391‰ [365,446]) than for LA (δ^13^C=24‰ [17,31]; *P*<0.001) and both were higher than for OA (δ^13^C=−4‰ [–7,–1]; *P*<0.001); all were higher than in the control group (δ^13^C=−21‰ [–22,–20]; *P*<0.001; *R*^2^_est_=0.38; Fig. 2B). The two species did not differ in feces δ^13^C for any FA (*P*=0.48) and maximum δ^13^C levels for OA and LA were not statistically different in any of the analyses.

**Fig. 3.**
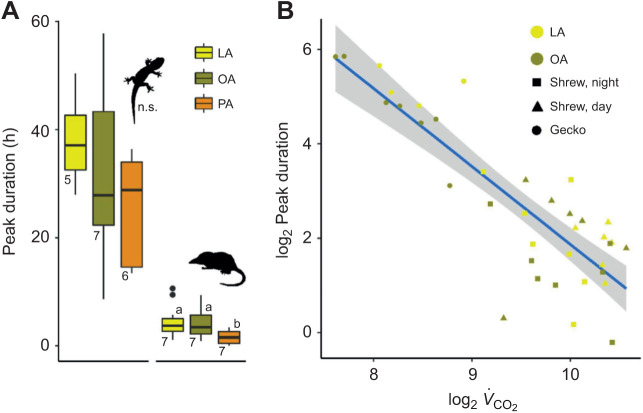
**Comparison of δ^13^C FA peak duration in geckos and shrews.** (A) Overall peak duration (i.e. the time from peak δ^13^C values to 25% of peak) did not differ between fatty acid groups. (B) Both within- and between-species differences in peak duration were mostly explained by metabolic rate, such that a doubling of *V̇*_CO_2__ (μl h^−1^) shortens the peak duration (h) by half. Blue line indicates GLM regression line with s.e.m. (shaded region).

The overall δ^13^C peak duration (i.e. the time from maximum δ^13^C values until δ^13^C values decreased to 25% of maximum), averaged 65±6 h in geckos and 5.5±0.4 h in shrews (mean±s.e.m.). δ^13^C peak duration did not differ between FAs in geckos (*P*>0.2; *R*^2^=0.09). In shrews, duration was 40% times shorter for PA than for OA (*P*=0.010) and 37% shorter for LA (*P*=0.001; *R*^2^_est_=0.14; Fig. 3A). Peak duration did not differ between day and night measurements in the shrews (*P*=0.976). For both species together, there was no difference between the peak duration of the OA and LA groups (*P*=0.177), but there was a negative correlation between peak duration and metabolic rate in both geckos (56.0% shorter peak duration per twofold increase in *V̇*_CO_2__; *P*=0.040) and shrews (45.7% shorter per twofold increase in *V̇*_CO_2__; *P*=0.018; *R*^2^_est_=0.89; Fig. 3B). Interestingly, the difference between the two species was not significant in both the intercept (*P*=0.486) and slope (*P*=0.646) of this correlation. OA and LA did not statistically differ from each other in any of the analyses.

**Fig. 4.**
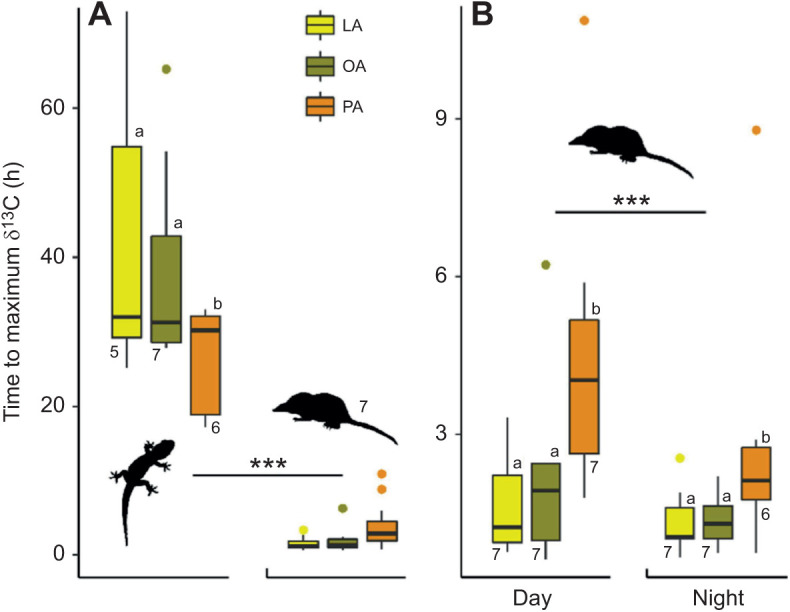
**Comparison of time to maximum δ^13^C FA levels in geckos and shrews.** (A) In geckos, the time from ingestion until peak δ^13^C was shorter for palmitic acid than for oleic and linoleic acid (*P*<0.05), whereas shrews reach peak δ^13^C ∼20 times faster than in geckos (*P*<0.001) for all FAs. (B) In shrews, the time from ingestion until peak δ^13^C was longer for palmitic acid than for oleic and linoleic acid (*P*<0.001) and shorter in night trials than day trials (*P*<0.001).

**Fig. 5.**
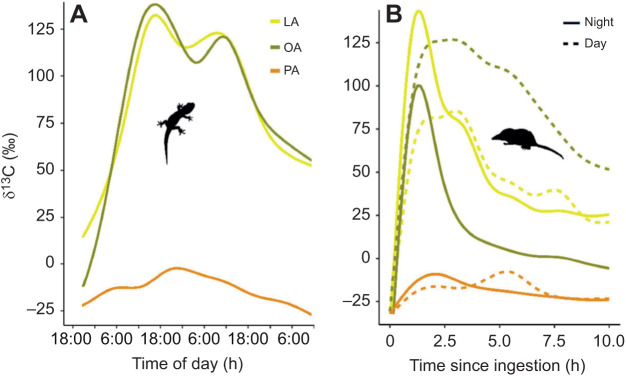
**δ^13^C trends during measurement (GAM smoothed) according to species, fatty acid group and day/night trial.** (A) In geckos, the twin δ^13^C peaks evident in both oleic and linoleic acid closely coincide with the end of subjective photophase (18:00). Three days of measurement are presented, restricted to hours during which all individuals (*n*=14, three shedding geckos excluded) were measured. (B) In shrews, the δ^13^C peak patterns for all fatty acids are faster and shorter in the night trials than in the day trials.

The mean time from ingestion until maximum δ^13^C values in geckos was shorter for PA (26±3 h) than for OA (42±7 h; *P*=0.015) and LA (38±9 h; *P*=0.020; Fig. 4A); for OA and LA, it was also weakly negatively correlated with metabolic rate (–20% per twofold increase in *V̇*_CO_2__; *P*=0.019 and *P*=0.027, respectively; *R*^2^=0.76). Although the geckos were in the dark and had ingested the ^13^C isotopically enriched crickets at different hours, they appear to have a rhythmic pattern of oxidation (Fig. 5A). The timing of maximum δ^13^C closely match the end of the subjective photophase (18:00 h) in both OA (18:20, 18:05, 18:06 and 16:45 h) and LA (18:06, 18:08, 18:13, 16:19 and 20:58 h), but not PA (07:42, 23:35, 06:15, 21:08 and 20:21 h). In shrews, the average time from ingestion until maximum δ^13^C did not correlate with metabolic rate but was 28.2% shorter in the night trials than in the day trials and was longer for PA (244 min [242,245]) than for OA (117 min [116,118]; *P*<0.001) and LA (103 min [101,103], *P*<0.001; Fig. 4B; Fig. 5B). Shrews had significantly shorter times between ingestion and maximum δ^13^C (*P*<0.001, *R*^2^_est_=0.48). Time to maximum δ^13^C for OA and LA did not statistically differ in any of the analyses. The full results are available in Table S1.

## DISCUSSION

Mediterranean house geckos (*H. turcicus*) and Etruscan pygmy shrews (*S. etruscus*) exhibited similar patterns of differential oxidation and egestion of the three ^13^C_1_ enriched FAs. While dietary OA (oleic acid, a MUFA) and LA (linoleic acid, a PUFA) were equally used as a metabolic fuel (evidenced by the similarity of δ^13^C peaks in the breath between FA treatments), the higher presence of LA in the feces of both species indicates that OA is more efficiently absorbed by the intestine. Dietary PA (palmitic acid, an SFA) had much lower oxidation rates and much higher egestion in the feces compared with OA and LA, indicating that although it can be used as a metabolic fuel, most of it is not, most probably owing to its inefficient uptake by the intestine. Several studies report lower oxidation of long- and medium-chain SFAs relative to MUFAs and PUFAs in humans (reviewed in Krishnan and Cooper, 2014), but also in other mammals (Leyton et al., 1987; Rosner and Voigt, 2018) and insects (Dikeman et al., 1981; Seltzer et al., 2023). This pattern is most commonly interpreted as preferential assimilation of SFAs in tissues over usage as a metabolic fuel, but our results raise the hypothesis that differential oxidation of an SFA primarily reflects differential absorption rather than differential oxidation or allocation to tissues. SFAs, and PA in particular, are known to be more slowly absorbed in the intestine, both in their uptake by the mucosa and the subsequent esterification and transportation (Ockner et al., 1972). Their physical properties, such as their high melting point and tendency to form insoluble calcium soaps in the alkaline environment of the intestine (Ramírez et al., 2001), can lead to much higher egestion, indicative of less FA available for uptake, transport and eventual β-oxidation in the cells. Recent findings in a study on bees complement our results: lower oxidation of PA compared with OA and LA, and also less allocation of PA to tissues (Seltzer et al., 2023), hinting at its higher egestion. A study in hibernating bats that found lower oxidation of PA than of LA of endogenous (rather than dietary) sources suggests that the faster transportation rates of PUFAs may also make them a preferable energy source (Rosner and Voigt, 2018). However, high allocation or absorption of SFAs is reported in a few studies on granivorous avians (McCue et al., 2011; Noyan et al., 1964; Tancharoenrat et al., 2014), which may represent adaptations for SFA absorption which are absent from insectivorous reptiles and mammals – but may also reflect methodological differences between the studies.

Trends of high PA egestion and its low oxidation were similar between the two species, despite the expectation that nutrient assimilation efficiency would be higher in animals with slower digestion (McConnachie and Alexander, 2004; Karasov and Martínez del Rio, 2007). Digestion is notoriously slower in reptiles compared with mammals, because reptiles must digest whole prey, and because reptiles have much lower metabolic rates to satisfy and therefore are not required to have fast digestion times to satisfy high energetic needs (Hulbert, 2003; Nagy, 2005). Our results may have missed the effect on nutrient assimilation since the lower body temperature of geckos affects digestion rates (which are temperature dependent; Pafilis et al., 2007) and FA fluidity, counteracting possible positive effects of a longer digestion time. Our results show that metabolic rate alone, not species, is the best predictor of the differences in peak duration (indicative of free FA depletion rates). The major difference between the two species was in the time it takes them to use the FAs, regarding both the time until peak δ^13^C values and the overall peak duration, both of which were more than tenfold longer in geckos. While the time until peak differed between FAs in both species, the PA rate was faster than OA and LA in geckos, but slower than OA and LA in shrews. This may result from two opposing influences: the higher saturation of PA slows its transport and oxidation rate, whereas its shorter chain length (16 instead of 18 carbons) leads to faster oxidation (Leyton et al., 1987). It is possible that within each species, a different factor weighs more heavily because different species digest over very different time scales.

While diel variation in the metabolic rate of reptiles is common (mainly but not exclusively in diurnal species: Angilletta, 2001; Dubiner et al., 2023; Hare et al., 2006;), this is an important documentation of a definite circadian rhythm of *V̇*_CO_2__ aligned with the activity phase. These rhythms are very similar to those found by Plasman et al. (2019) in the lizard *Agama atra* and were likewise present during digestion, showing that lizard SDA is an oscillatory phenomenon, making it challenging to calculate here without good data on pre-feeding oscillations (McCue, 2006). Importantly, we also present rudimentary evidence of a circadian rhythm of FA oxidation in geckos, which may indicate one of two things. Most probably, the decrease in δ^13^C concomitantly with the animal's increased metabolic rate represents shifts in the energy source toward endogenous reserves (less rich in ^13^C). Alternatively, as there is ample evidence that the cell-autonomous circadian clock controls mitochondrial oxidative function in mammals (Bray and Young, 2011; Gooley, 2016; Peek et al., 2013), our results could hint at a similar pattern in reptiles. δ^13^C values for OA and LA reached a peak concurrently with the onset of the subjective scotophase (their activity time), in accordance with studies showing that the peak capacity of FA oxidation-related RNA transcription in the mitochondria is at the beginning of the active phase (Bray and Young, 2011; Peek et al., 2013). In shrews, the measurement duration was too short to determine the presence or absence of circadian rhythms, but the significant difference in rate (i.e. time until peak) between day and night trials suggest the presence of such rhythms.

Differences in the uptake and subsequent utilization of different FAs carry implications for studies on the effects of dietary FA composition. Primarily, these differences challenge the isocaloric assumption of experimental diets, which have been used in the past to show changes in the physiology of reptiles (Simandle et al., 2001) and mammals (Ayre and Hulbert, 1996; DeLany et al., 2000; Ruf et al., 2006), among other taxa. However, there are some caveats to our conclusions that must be considered. Firstly, the identical patterns between geckos and shrews were produced by different quantities of FAs in the treatment (1μl and 5 μl, respectively), so we cannot rule out a different outcome had the quantities been high for both species. Moreover, FAs administered in laboratory conditions potentially do not have the same properties that they would in the animal's natural diet, affecting absorption. Most animals do not consume pure free FAs in large amounts, ingesting most of their lipids as more complex phospholipids and triglycerides. Additionally, PA was shown to be better absorbed by mammals when it is in the *sn*-2 position of the glycerol backbone (Ramírez et al., 2001; however, that position occurs in milk and would not commonly be present in the diet of adult insectivores). FA absorption can also be influenced by the physical environment in the intestine and the context and relative quantity of other FAs in the diet (Thomson et al., 1989; Tancharoenrat et al., 2014) or the presence of other substances, such as calcium ions (Tomarelli et al., 1968). Artificial ways of improving the FA administration, such as emulsifying the solid insoluble PA might also have improved its absorption (Levin et al., 2017). However, in a recent study on bumblebees (Seltzer et al., 2023), emulsification of the PA did somewhat improve its allocation to tissue, but did very little to change the overall pattern, lending support to the results presented here. Therefore, although changes in the method of FA administration may lead to different results, we encourage future nutritional studies on FAs to account for, or at least address, the risk of bias by differential absorption.

### Conclusions

Long chain saturated fatty acids from the diet are used less as metabolic fuel (and at a different rate) than mono- and polyunsaturated fatty acids in geckos and shrews. This may simply reflect a difference in intestinal absorption, rather than a cellular ‘triage’ of fatty acids for oxidation or allocation in tissues, as is commonly theorized. Ingested fatty acids are processed at a faster rate in shrews than in geckos and this is well explained by differences in their metabolic rates. Geckos exhibit a circadian rhythm of both metabolic rate and dietary fatty acid usage; shrews exhibit different usage patterns between day and night trials. The very similar patterns between species so phylogenetically distinct as *H. turcicus* and *S. etruscus* hints at a conserved function across vertebrates, but since they are similar in body mass and diet, it is necessary to validate these results in other (large, diurnal, herbivorous) species, and in other vertebrate groups (birds, amphibians, fish). Additional fatty acids of different lengths, unsaturation levels and concentrations, as well as manipulating body or ambient temperatures, should also be explored.

## Supplementary Material

10.1242/jexbio.245963_sup1 Supplementary information

Table S1.
