# Supplementary material for: Patterns of fatty acid usage in two nocturnal insectivores: the Mediterranean house gecko (Hemidactylus turcicus) and the Etruscan pygmy shrew (Suncus etruscus)
Source: J Exp Biol. 2023 Oct 11;226(19):jeb245963. doi: 10.1242/jeb.245963 (PMC10656425; doi:10.1242/jeb.245963)
Supplement: Supplementary information [file jexbio-226-245963-s1.pdf]

Table S1.

Available for download at
https://journals.biologists.com/jeb/article-lookup/doi/10.1242/jeb.245963#supplementary-data

Journal of Experimental Biology • Supplementary information
